# Analysis of Kinematic and Muscular Fatigue in Long-Distance Swimmers

**DOI:** 10.3390/life13112129

**Published:** 2023-10-27

**Authors:** Luca Puce, Carlo Biz, Alvise Ruaro, Fabiana Mori, Andrea Bellofiore, Pietro Nicoletti, Nicola Luigi Bragazzi, Pietro Ruggieri

**Affiliations:** 1 Department of Neuroscience, Rehabilitation, Ophthalmology, Genetics, Maternal and Child Health (DINOGMI), University of Genoa, 16132 Genoa, Italy; luca1puce@gmail.com; 2 Orthopedics and Orthopedic Oncology, Department of Surgery, Oncology and Gastroenterology (DiSCOG), University of Padova, 35128 Padova, Italy; alvise.ruaro@aopd.veneto.it (A.R.); fabiana.mori@studenti.unipd.it (F.M.); andrea.bellofiore@studenti.unipd.it (A.B.); pietro.ruggieri@unipd.it (P.R.); 3 Department of Neurosciences, University of Padova, 35128 Padova, Italy; pietronicoletti.ft@gmail.com; 4 Laboratory for Industrial and Applied Mathematics (LIAM), Department of Mathematics and Statistics, York University, Toronto, ON M3J 1P3, Canada

**Keywords:** electromyography, task failure, swimming, muscle coordination

## Abstract

Muscle fatigue is a complex phenomenon that is influenced by the type of activity performed and often manifests as a decline in motor performance (mechanical failure). The purpose of our study was to investigate the compensatory strategies used to mitigate mechanical failure. A cohort of 21 swimmers underwent a front-crawl swimming task, which required the consistent maintenance of a constant speed for the maximum duration. The evaluation included three phases: non-fatigue, pre-mechanical failure, and mechanical failure. We quantified key kinematic metrics, including velocity, distance travelled, stroke frequency, stroke length, and stroke index. In addition, electromyographic (EMG) metrics, including the Root-Mean-Square amplitude and Mean Frequency of the EMG power spectrum, were obtained for 12 muscles to examine the electrical manifestations of muscle fatigue. Between the first and second phases, the athletes covered a distance of 919.38 ± 147.29 m at an average speed of 1.57 ± 0.08 m/s with an average muscle fatigue level of 12%. Almost all evaluated muscles showed a significant increase (*p* < 0.001) in their EMG activity, except for the *latissimus dorsi*, which showed a 17% reduction (ES 0.906, *p* < 0.001) during the push phase of the stroke cycle. Kinematic parameters showed a 6% decrease in stroke length (ES 0.948, *p* < 0.001), which was counteracted by a 7% increase in stroke frequency (ES −0.931, *p* < 0.001). Notably, the stroke index also decreased by 6% (ES 0.965, *p* < 0.001). In the third phase, characterised by the loss of the ability to maintain the predetermined rhythm, both EMG and kinematic parameters showed reductions compared to the previous two phases. Swimmers employed common compensatory strategies for coping with fatigue; however, the ability to maintain a predetermined motor output proved to be limited at certain levels of fatigue and loss of swimming efficiency (Protocol ID: NCT06069440).

## 1. Introduction

Muscle fatigue is a complex and multifactorial phenomenon that is closely related to the characteristics of the motor activity being performed [1,2]. In high-intensity activity, fatigue is commonly observed as a reduction in a muscle’s capacity to generate or sustain force over a period of time, a phenomenon known as “mechanical failure” [3,4]. This term refers to the point at which muscle tissue undergoes structural and functional changes impairing its ability to contract efficiently [5]. However, fatigue can also occur in the absence of mechanical failure, particularly at submaximal intensity levels of activity. In such cases, the neuromuscular system employs compensatory strategies at various levels to delay the onset of fatigue until task failure [6], allowing for prolonged motor activity.

Numerous studies have demonstrated changes in both motor unit recruitment and the coding rate (i.e., the rate at which spinal motor neurons discharge action potentials) during strenuous physical activity [4,7]. A growing body of research has identified alternating levels of muscle activity among synergistic muscles during low-level submaximal isometric exercise [6,8] or during a constant-load rowing exercise performed until task failure [9]. In addition, changes in muscle coordination and timing have been reported during dynamic tasks such as pedalling [10,11], jumping [12], vertical jumping [13], and swimming [14,15,16,17]. Despite extensive research, however, little is known about muscle compensation strategies during cyclic activities such as swimming, where the unique nature of the sport requires synergistic interactions among trunk, upper, and lower limb muscles for propulsion [18]. Unlike other cyclical activities, such as running and cycling, where the arms primarily stabilise the trunk without contributing to propulsion, swimming may provide a greater opportunity to utilise intermuscular compensation strategies. 

As the upper limbs play a key role in propulsion in water [19], the effect of fatigue on kinematic parameters has been studied, with a focus on arm action [20,21]. In all four competitive strokes (butterfly, backstroke, breaststroke, and freestyle) and, generally, in races of 200 m and longer, fatigue leads to changes in stroke frequency (SF—time required to complete a stroke cycle) and stroke length (SL—distance travelled during each stroke cycle), thus affecting the swimmer’s ability to maintain rhythm [22,23]. In addition, longer propulsive phases and shorter recovery phases in the stroke cycle have been shown to improve performance [24,25,26,27]. 

The study of the muscles involved in swimming through electromyographic (EMG) analysis has helped deepen our understanding of how athletes maintain peak performance even in the face of muscle fatigue [23]. Recent research [28,29,30,31,32,33,34,35] has revealed the central role of specific muscles in maintaining speed and performance under fatigue conditions. For example, the *latissimus dorsi* (LD), *triceps brachii* (TB), and *pectoralis major* (PM) muscles often exhibit dynamic and strategic adaptations [30,32]. As fatigue sets in, their EMG activity progressively increases, effectively compensating for the decreased efficiency of other muscle groups [29]. 

Despite the wealth of research on the subject, the evaluation of the compensation mechanisms put in place by the neuromuscular system to delay mechanical failure remains challenging. To better understand how the body adapts to prolonged, strenuous exercise, it is important to observe athletes as they maintain a controlled, steady pace over a given time period [12]. In this way, if there are no speed changes during the activity, any neuromuscular adaptations due to fatigue can be attributed to compensatory strategies [12]. Unfortunately, in the existing scholarly literature, most studies used uncontrolled speed tasks, and, as such, the analysis was carried out without distinguishing between the phase where the athlete’s performance is constant and the phase where mechanical failure takes over, which does not allow a completely correct understanding of the phenomenon under study. In the latter situation, the compensatory mechanisms have lost their effectiveness.

Therefore, our aim in this study was to investigate in detail the compensatory mechanisms used by swimmers during the performance of a controlled speed swimming test. Specifically, we proceeded to collect and analyse kinematic parameters of the upper limb, as well as to observe the EMG activity related to twelve muscle groups involved in swimming propulsion in distinctive phases. These phases include the start of the test under non-fatiguing conditions, the moment before the inability to maintain the prescribed speed, and the phase of mechanical failure. 

We hypothesised that, as fatigue becomes more pronounced and the point of the inability to maintain a predetermined speed is approached, increased EMG activity will occur in key muscles, while other muscle groups may show more obvious signs of fatigue. In addition, changes in the rhythm and coordination of upper limb movements may occur.

## 2. Materials and Methods

### 2.1. Recruitment of Participants 

The study protocol was reviewed and approved by the local ethics committee of the University of Genoa, Genoa, Italy (protocol number 2020/21). Participation in the study was subjected to the written, informed consent of the participant and his/her parent or guardian. This basic procedural requirement ensured that all parties involved were fully informed and in agreement regarding the individual’s commitment to the research. 

Twenty-one swimmers (fifteen males and six women, mean age 19.57 ± 2.64 years, height 178.62 ± 7.14 cm, weight 69.29 ± 7.94 kg, World Aquatic Points Scoring 780.57 ± 53.48 based on the swimmers’ best official performance on the 1500 m long course) were carefully selected from a local prestigious team composed of the most skilled swimmers from Liguria (Italy). Their average annual water training volume was 17.58 ± 3.60 h per week, equivalent to a distance of 57.38 ± 12.91 km. The weekly training hours on land corresponded to 3.00 ± 0.95 h. Table 1 shows their physical and athletic characteristics. Each swimmer was carefully selected based on strict eligibility criteria, including (i) middle- or long-distance swimmer specialising in the front crawl, (ii) at least 3 years of experience in international competition, (iii) daily use of flashing light for pace control in aerobic and anaerobic threshold and maximum oxygen consumption training. The only exclusion criterion was the presence of muscle pain or soreness that could prevent the athlete from performing at their best.

### 2.2. Experimental Design 

First, each athlete performed a personalised warm-up on land (≈30 min of stretching and rotator cuff exercises with elastic bands) and in the water (≈15 min of low-intensity aerobic swimming, ≈10 min of drills, and ≈10 short sprints for a total distance of ≈1500 m). Next, participants were asked to perform a swimming test in an indoor 50 m Olympic pool with a water temperature of ≈27°. The task was to swim for as long as possible while maintaining an average speed equal to the personal record for the 1500 m front crawl. If the athlete was no longer able to keep up, he/she had to swim at his/her maximum power for another ≈15 m. A yellow flashing underwater lighting system (Virtual Swim Trainer, distributed under licence by Indicotech s.r.ls., Turin, Italy) was installed at the bottom of the pool to help the athlete maintain the required speed. The device consists of a 45 cm LED light spot (20 LEDs) that extends from the beginning to the end of the pool (50 m). Because of the measurement equipment attached to the body (electrodes and markers for kinematic analysis), an underwater turn was allowed, but a dive start was not. 

**Figure 1:**
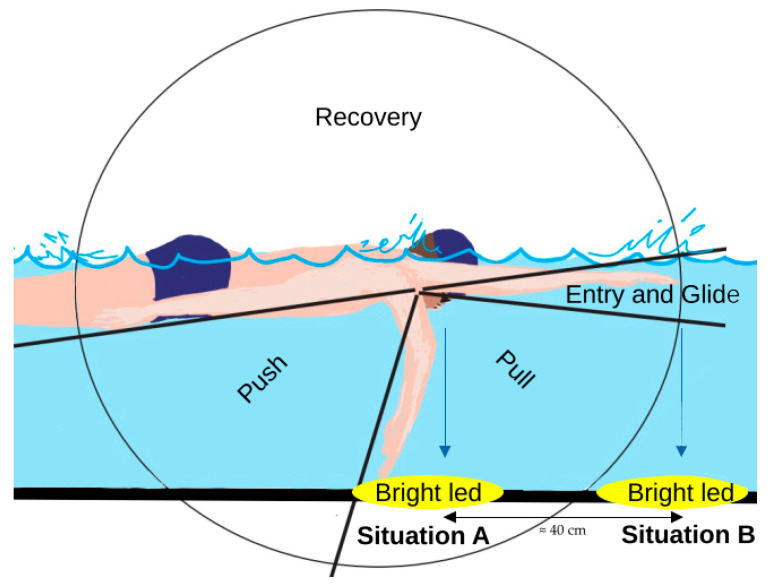
The figure illustrates the conditions under which the swimmer is involved in the study. In situation A, corresponding to the non-fatiguing and pre-mechanical failure phases, the swimmer’s face is perpendicular to the flickering light used for rhythm control. In situation B, representing the mechanical failure phase, the swimmer’s face is no longer aligned with the flashing light. In addition, the different phases of the stroke cycles are outlined.

We performed kinematic and EMG analysis (specific for all parts of the stroke cycle) during three different swimming phases within the task, namely, the non-fatigue phase (the first three strokes from the start of the test immediately after the underwater swim), the pre-mechanical failure phase (the last three strokes before the inability to maintain the set speed), and the mechanical failure phase (referring to the three strokes in which the inability to maintain the set speed occurs). The first two phases are characterised by the perpendicularity of the swimmer’s face to the flashing light used for rhythm control. In the last phase, the swimmer’s face is no longer perpendicular to the flashing light, as can be seen in Figure 1. If the strokes considered for analysis were interrupted by the turn phase, we considered the stroke(s) before the turn and the stroke(s) after the end of the underwater swim. In addition, a further analysis was carried out on the evolution of the kinematic and EMG parameters in the sections corresponding to 25%, 50%, 75%, and 100% of the distance travelled between the non-fatigue and pre-mechanical failure phases.

### 2.3. Kinematic Assessment 

The outcome measures included several kinematic parameters, such as swimming speed (m/s) and distance (m) travelled at the target speed. In addition, within the three different stroke cycles of the three phases (no fatigue, pre-mechanical failure, and mechanical failure), SF (cycles·min^−1^), SL (m·cycle^−1^), stroke index (SI; m^2^·s^−1^·cycles^−1^), and the EMG activity of different parts of the stroke cycle were examined.

The athlete’s swim speed was optimised using Virtual Swim Trainer software linked to a flashing underwater lighting system. Using parameters such as distance from the wall to the turning point, distance travelled, and time spent underwater swimming (previously recorded for all athletes), the device’s software allowed the pace settings to be adjusted to include the acceleration phases that occur during the initial push off the wall and in the phases following the turn.

The swimming trials were filmed in the sagittal plane with two cameras (GoPro Hero 8 model, GoPro, San Mateo, CA, USA) at a 50-frames-per-second acquisition rate, one above the water surface (height above the surface: 150 cm at an angle of 30 degrees) and one below (height below the surface: 50 cm at an angle of 0 degrees). The cameras were mounted on a pushcart that was manoeuvred by two operators. This trolley moved along the edge of the pool at the same speed as the swimmers, and not at the set speed [30]. In addition, the pushcart was positioned ≈4 m away from the swimmer. This arrangement allowed the underwater camera to fully capture both the flashing underwater lighting system and the submerged part of the athlete’s body. Synchronisation of the two cameras was achieved by means of a diode flashlight visible from both cameras [31] before the underwater camera was put in the water. The video recording was manually synchronised with the EMG trace by touching the EMG sensor, which is visible in both the video and the EMG trace [34]. 

Markers were attached to the participants’ joints for kinematic analysis and to distinguish different parts of the stroke cycle. Specifically, the propulsion phase began when the outstretched arm entered the water, and the “entry and glide” phase continued with the “pull” phase and ended when the arm left the pelvis above the water, corresponding to the end of the “push” phase and the beginning of the “recovery” phase [17]. Figure 1 shows these different phases in detail. 

SF was calculated based on the time taken to complete three strokes, while SL was determined by dividing speed by SF. SI was calculated as the product of speed, and SL and is considered a valid indicator of swimming efficiency [36,37]. The equations for reporting these parameters are as follows:

(1) $$\mathrm{SF}\;[\mathrm{cycles}\cdot \min^{-1}]=\frac{3}{(Time,in\;second,to\;complete\;3\;stroke\;cycles}\times 60$$

(2) $$\mathrm{SL}\;[m\cdot {\mathrm{cycle}}^{-1}]=\frac{Swimming\;speed}{SF}$$

(3) $$\mathrm{SI}\;[m^{2}\cdot s^{-1}\cdot {\mathrm{cycles}}^{-1}]=Swimming\;speed\times SL$$

### 2.4. EMG Assessment 

The amplitude (Root Mean Square, RMS (mV)) and the median frequency of the EMG power spectrum (MDF (Hz)) of the EMG signal were chosen as outcome measures. The latter assessment allowed us to study the manifestations of electrical fatigue from the beginning of the task to the end of the second phase.

Bipolar surface electrodes were placed on the right side of the body covering twelve different muscles: *Flexor Carpi Radialis* (FCR), PM *pars clavicularis*, TB *caput lateralis*, *Biceps Brachii* (BB), LD, *Superior Trapezius* (ST), *Rectus Femoris* (RF), *Biceps Femoris* (BF), *Gastrocnemius Medialis* (GM), *Erector Spinae* (ES), *Tibialis Anterior* (TA), and *Deltoideus Lateralis* (DL) according to the “Surface Electromyography for Non-Invasive Assessment of Muscle” (SENIAM) guidelines for electrode placement [30]. The GM and TA muscles were subsequently excluded from the analysis because their EMG signals were attributed to movement artefacts rather than motor unit potentials. Prior to electrode fixation, the skin surface was shaved and cleaned with alcohol. The electrodes were placed parallel to the direction of the muscle fibres in the middle of the contracted muscle belly. Waterproof adhesive tape (Fixomull transparent, BSN medical, Hamburg, Germany) was applied to avoid changes induced by underwater recording [38]. These muscles were chosen because previous research has shown their importance in swimming propulsion [18]. 

The EMG signal was acquired using a wireless waterproof EMG device (Cometa Srl, Milan, Italy) with a first-order bandpass filter in the range of 10–500 Hz and digitalised at 2000 samples/s. The electrical signals produced by the muscles were processed according to specific guidelines. The signals were filtered at a specific range (20–450 Hz), then rectified, smoothed with a low-pass filter (5 Hz, 4th order Butterworth), and finally normalised with respect to the peak activity obtained from the reference signal (a method called “dynamic peak”) [39]. To assess the manifestations of electrical muscle fatigue, the EMG spectrum was estimated within a window of 480 samples, ranging from the end of the pulling phase to the beginning of the pushing phase. The Fast Fourier Transform (FFT) function was used to calculate a linear magnitude FFT on the motion of the selected data segments. The MDF was identified as the harmonic corresponding to the 50th percentile of the energy distribution in the frequency spectrum. Open-source Python software distributed by Anaconda Inc. was used to perform these calculations. The resulting analysis produced a graph showing the values of MDF over time. To evaluate the temporal evolution of MDF, a linear fit of the data set was performed, and the slope (temporal slope of MDF) was derived. These slopes were then normalised to the value of the regression line at the start time of the first activation interval, analysed, and expressed as percentages. A gradual decrease in MDF during exercise indicates the presence of muscle fatigue [40]. 

Finally, each activation interval was averaged by interpolation, resulting in 101 points for both the propulsion and recovery phases. In addition, the time of each phase was normalised so that each represented 50% of a stroke cycle. The propulsive phase ranges from 0% to 50%, and the recovery phase ranges from 51% to 100%.

### 2.5. Statistical Analysis 

Summary statistics were generated by calculating the means and standard deviations for each of the parameters under investigation. Due to the limited sample size employed, the normality of data distribution underwent examination through the Shapiro–Wilk test. Based on the test results, comparisons were made using repeated-measure analysis of variance (rmANOVA) or Friedman’s analysis of variance by ranks. Pairwise comparisons were also carried out. These analyses aimed to identify any differences in the variables under study among different conditions (i.e., non-fatigue, pre-mechanical failure, and mechanical failure), as well as among different phases (i.e., entry and glide, pull, push, and recovery). 

Depending on the type of analysis, the effect size (ES) was computed using partial eta squared, wherein ES values of 0.01, 0.06, and 0.14 were deemed small, medium/moderate, and large [40], respectively, or Kendall’s W, wherein ES values < 0.10 were considered very small, with 0.10–0.29 considered small, 0.30–0.49 considered moderate, and ≥0.5 considered large [41,42]. For either paired or independent pairwise comparisons, the rank-biserial correlation was computed [43], the interpretation of which is similar to Kendall’s W [42].

Furthermore, data were reported for the overall population and broken down according to sex/gender and swimming distance specialisation (long-distance and middle-distance swimmers). 

All statistical analyses were conducted using the commercial software “Statistical Package for Social Sciences” (SPSS for Windows, version 28.0, IBM, Armonk, NY, USA) and the open-source Jamovi software (version 2.3, The Jamovi Project, 2022, https://www.jamovi.org (accessed on 12 May 2023)). Findings reaching a significance threshold of 0.05 were considered statistically significant.

## 3. Results

### 3.1. Kinematic Parameters

Between the non-fatigue phase and the pre-mechanical failure phase, the athletes covered a distance of 919.38 ± 147.29 m at an average speed of 1.57 ± 0.08 m/s. In the mechanical failure phase (≈15 m), the speed decreased to 1.41 ± 0.10 m/s. Table 2 shows the results of the kinematic analysis, focusing on the changes between the non-fatigue phase and both the pre-mechanical failure and mechanical failure phases.

In the first comparison of kinematic parameters between the non-fatigue phase and the pre-mechanical failure conditions, a reduction in SL was observed, amounting to a decrease of 6% (ES 0.948, *p* < 0.001, large effect), which was counterbalanced by an increase in SF of 7% (ES −0.931, *p* < 0.001, large effect). Notably, SI also decreased by 6% (ES 0.965, *p* < 0.001, large effect). In the mechanical failure phase, all parameters analysed showed a uniform decrease of 15% (ES 1.000, *p* < 0.001, large effect), 6% (ES 0.827, *p* < 0.001, large effect), and 5% (ES 0.883, *p* < 0.001, large effect) for SI, SL, and SF, respectively. 

### 3.2. EMG Parameters

**Figure 2:**
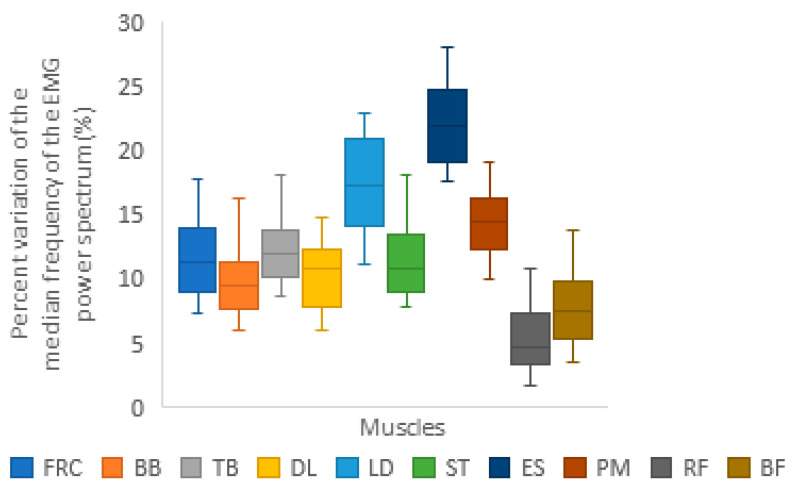
The figure shows the temporal evolution of the electrical manifestations of fatigue from the initial moment of the test (non-fatigue phase) to the moment immediately preceding the loss of the ability to maintain the predetermined speed (pre-mechanical failure). Abbreviations: FCR = *Flexor Carpi Radialis*; BB = *Biceps Brachii*; TB = *Triceps Brachii caput lateralis*; DL = *Deltoideus Lateralis*; LD = *Latissimus Dorsi*; ST = *Superior Trapezius*; ES = *Erector Spinae*; PM = *Pectoralis Major pars clavicularis;* RF = *Rectus Femoris*; BF = *Biceps Femoris*.

Figure 2 presents the temporal evolution of the electrical manifestations of muscle fatigue, ranging from the non-fatigue phase to the pre-mechanical failure phase. In the upper limbs, fatigue levels of 12%, 8%, 12%, and 10% were observed in FRC, BF, TB, and DL, respectively. The trunk showed 11% fatigue in ST, followed by 14% in PM, 17% in LD, and 22% in ES. In the lower limbs, RF showed a fatigue rate of 5%, while BF showed a fatigue rate of 8%.

**Figure 3:**
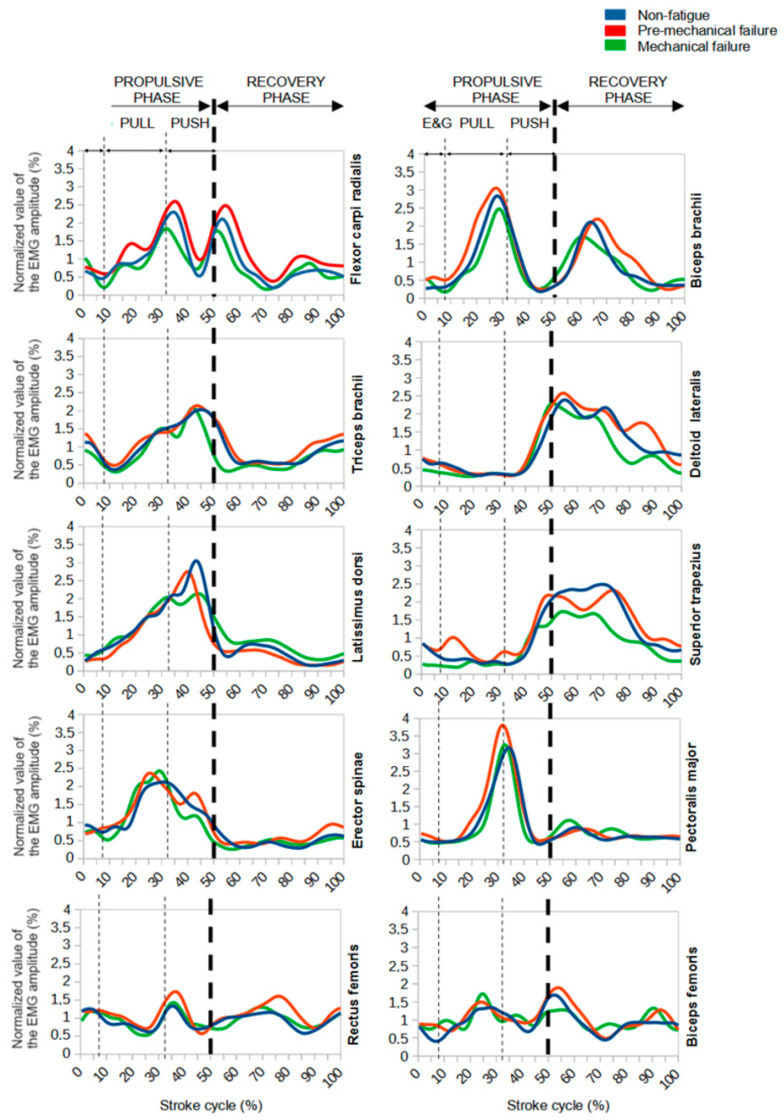
Comparison of muscle profiles at 100% of a stroke cycle across different phases and muscles studied. Data were obtained by calculating the average muscle profile of all participants.

Table 3 and Figure 3 provide a detailed analysis of the variations in EMG activity during different phases of the stroke cycle. Specifically, these changes are observed between the non-fatigue phase and both the pre-mechanical failure and mechanical failure phases.

During the non-fatigue and mechanical pre-fatigue phases of upper limb muscles, there was a significant increase in activity in all phases studied, except for the push phase in the BB and TB muscles (0% and 1%, very small and small effects, respectively). For trunk muscles, a significant increase was noted during the pulling phase in all muscles examined, from 7% for LD (large effect) to 49% for PM (large effect). As for the pushing phase, the results showed variations. PM and ST showed an increase (4%, small effect, and 23%, large effect, respectively), although only the latter was found to be statistically significant, while ES maintained a constant level (0%, very small effect) and LD decreased (−17%, large effect). Looking at the lower limb muscles, there was an increase in muscle activity, except in the E&G phase for the RF muscle (−2%, moderate effect). Comparing the non-fatigue phase with the point of mechanical failure, there was a general trend of decreasing muscle activity, except for the upper limb muscles, where activity remained essentially stable. 

Kinematic activity and EMG values did not differ between men and women, nor between middle- and long-distance specialists (Table 4).

Table 5 shows the analysis of EMG activity and kinematic changes expressed as percentage changes at specific intervals during the activity. These intervals include 25%, 50%, 75%, and 100% of the distance travelled between the non-fatigue and pre-mechanical failure phases.

## 4. Discussion

The purpose of this study was to examine strategies for compensating for fatigue with a particular focus on the ability to maintain a predetermined pace at a consistent speed over an extended period. As hypothesised, between the non-fatigue and pre-mechanical failure phases, the swimmers experienced muscle fatigue that resulted in changes in the rhythm and coordination of upper limb movements. Specifically, there was a decrease in SL and stroke efficiency (SI), compensated by an increase in SF. EMG activity also followed the initial hypothesis, with increased activity of almost all muscles, while the LD muscle showed the opposite behaviour. On the other hand, the inability to maintain a detectable rhythm (mechanical failure phase) was attributed to a decrease in all of the kinematic and EMG parameters analysed, with the exception of the lower limbs. Finally, no significant differences in fatigue coping mechanisms were observed between genders or between medium- and long-distance specialists.

### 4.1. Electrical Manifestations of Muscle Fatigue 

Higher levels of fatigue (on average, up to two times) were observed in the upper body than in the lower body. This finding is consistent with previous studies [31,37]. The authors attributed their results to the fact that leg action plays a relatively minor role (about 10%) in propulsion [19]; its primary effect is to improve buoyancy and thereby reduce water resistance (drag). For the upper body, fatigue levels ranging from 8% to 22% were found. Although our swim test differs significantly in distance (approximately 900 m), the fatigue levels are similar to those observed for much shorter tasks. For example, Stirn et al.’s [30] and Puce et al.’s [40] studies reported fatigue levels of 20% to 25% and 10% to 25%, respectively, for a 100 m front-crawl task. Even in a study involving a distance of 200 m, fatigue levels did not exceed 20% [35]. Therefore, it appears that fatigue levels are independent of task length but dependent on intensity. In all of these studies, athletes were instructed to maintain the highest possible speed relative to the distance to be covered.

### 4.2. Muscle Activity 

During fatigue, there is a significant increase in EMG activity in almost all of the muscles involved between the first and second phases. The progressive increase stopped when a certain threshold of overall fatigue was reached, defined as the “mechanical failure point” [44]. This point corresponded to approximately 12% of the average assessed muscle fatigue. However, it should be stressed that this is only a general view, as various factors, such as synergistic muscle interactions between agonists and antagonists, can limit the contractile capacity of a given muscle, even if it is not completely exhausted [45].

While there is a similar EMG increase in all examined muscles, FCR, BB, and PM exhibit significantly higher values. Rouard and Billat [46] investigated the EMG activity of six upper extremity muscles, noting that BB, PM, and FRC play pivotal roles in the swimmer’s propulsive phase, particularly when the hand contacts the water and during the initial stages of the stroke. Similar results were found in studies by Pink et al. [47] and Rouard et al. [48]. Additionally, it was determined that the action of FCR is crucial for wrist stabilisation, enhancing the capacity to generate force among high-level swimmers [49].

This increase in activity of almost all muscles examined, which does not affect speed, is clearly due to fatigue management strategies [7]. It can, therefore, be concluded that the additional recruitment of motor units was not aimed at increasing mechanical force production, but rather at counteracting muscle fatigue [50,51]. 

Physiologically, this strategy can be explained by Henneman’s size principle [36,37], which has been observed in numerous studies during submaximal intensity-controlled tasks [52]. Specifically, according to this principle, motor units are recruited in an increasing order of size during muscle contraction. This means that smaller motor units are activated first in response to a mild neural stimulus or to perform low-intensity muscle tasks. As the force required increases, as in the case of fatigue, as shown in our study, the central nervous system begins to recruit medium-sized motor units and finally large motor units to provide the required force. In the third phase, however, a decrease in EMG activity is observed in all muscles. This can be explained by the fact that maximum motor recruitment has been reached. From this point on, fatigue predominates and cannot be compensated. This suggests that the ability of the nervous system to provide adequate activation of the muscles involved in the specific task is closely related to the ability to maintain a predefined motor output, such as a given swimming speed [53]. 

From this perspective, the behaviour of the LD muscle is counterintuitive, as it shows a decrease in muscle activity. It is difficult to attribute this trend to an inability to recruit new motor units, but it seems more likely that it is the main muscle involved in propulsion [54] and is already active at maximum capacity even at submaximal speeds. In contrast, the *pectoralis* is the muscle that shows a significant increase in its activity. This is in line with other studies on swimming that highlight its importance in rhythm management under fatigue conditions [29]. However, there are several challenges in comparing the above findings with the existing literature. First, the most recent studies include different distances and muscles. This leads to inhomogeneous results. In addition, EMG activity was assessed in other studies under uncontrolled speed conditions. This provides a broad perspective on performance rather than specific insights into compensation strategies. 

For example, Ikuta et al. [29] assessed the relationship between performance and the muscle activity of 11 muscles in 20 elite swimmers during an all-out test (4 × 50 front crawl). A decrease in performance was found to correlate with a decrease in muscle activity (excluding the *pectoralis* muscle) during the test. Interestingly, the *pectoralis* muscle showed a progressive increase in amplitude during the test, leading the researchers to believe that it may play a crucial role in maintaining swimming speed under fatigue conditions. In their study, Stirn et al. [30] analysed the muscle activity of 11 experienced swimmers during a 100 m front crawl. The results showed that the pectoral muscles and the upper LD showed no significant changes, while the TB and the lower LD showed an increase in amplitude. In addition, the duration of muscle activation related to the stroke cycle decreased only for the *pectoralis* muscle. More recently, Figueiredo et al. [31] found that the muscle activity of FCR, BB, TB, PM, and upper *trapezius* increased during specific phases of the stroke during a 200 m full front crawl in 10 world-class male swimmers. However, the level of leg muscle activity remained constant. 

In conclusion, patterns of muscle activity in different muscles during an all-out front-crawl test vary between studies, with some showing significant changes and others not. These discrepancies suggest that multiple factors, including individual differences, stroke phases, and swimming distance, may contribute to the observed differences.

### 4.3. Kinematic Evaluation 

A consistent pattern emerges during the fatigue process (between non-fatigue and pre-mechanical failure). A decrease in SL is observed, resulting in a compensatory increase in SF. This phenomenon has been well documented [22,23]. Typically, as muscle strength decreases with fatigue, swimmers struggle to maintain their initial SL. Their response to this decrease is to increase their SF. However, as fatigue progresses, swimmers eventually reduce their SF. This leads to a decrease in swimming speed [30]. Our results provide further support for this observation, which was evident during the mechanical failure phase, where both SF and SL decreased, resulting in a corresponding decrease in speed. 

An important finding of this study is that the compensation strategy remains effective up to a certain threshold of swim efficiency (assessed by means of SI), which is set at −6%. Beyond this threshold, the compensation strategies lose their effectiveness. However, even in this scenario, it is difficult to accurately assess the interaction between kinematic and EMG parameters. Determining the extent to which one aspect predominates over the other is also challenging. 

Looking at muscle fatigue in relation to kinematic parameters, the most fatigued muscle is not the one responsible for propulsion, but rather the one responsible for maintaining body position in the water, the ES muscle. Working in synergy with the *rectus abdominis*, this muscle allows the swimmer to minimise water resistance. Consequently, a decrease in contractile capacity due to significant fatigue can lead to a deterioration in body position, resulting in a decrease in SL, a loss of efficiency, and an increased demand for energy from other muscles to maintain the established pace [55]. Indeed, the ability to generate efficient propulsion while reducing water resistance [56] determines a swimmer’s speed. These hypotheses need to be confirmed by analysing endurance in relation to fatigue. 

The lack of differences between middle- and long-distance swimmers for each of the outcomes assessed is an expected result. Despite differences in training volume [57], both groups train daily with similar goals and race strategies, such as increasing aerobic endurance and maintaining a steady pace [58]. However, the more surprising finding is the lack of gender differences. Specifically, men were found to have significantly greater underwater torque than women [59]. However, women were still found to have a higher SI [60]. This greater efficiency in women can be attributed to several factors, including their smaller body size (resulting in less drag) [61], higher body fat percentage (resulting in less body density) [59], and shorter leg length (resulting in a more streamlined posture) [61]. Together, these components (strength in men and anthropometric characteristics in women) contribute to subtle differences between the two sexes [62], resulting in the use of relatively similar compensation strategies.

## 5. Conclusions and Practical Applications

Our comprehensive analysis of the EMG and kinematics of swimmers performing a task at a controlled speed provided valuable insights into the mechanisms used to cope with fatigue. From a kinematic point of view, we observed a significant decrease in SL, which was well compensated for by an increase in SF. On the EMG front, our results showed a general increase in muscle activity, indicating that the ability to maintain a predefined speed is closely related to the ability of the nervous system to provide adequate activation. No differences in the mechanisms used to cope with fatigue were found between men and women or between medium- and long-distance specialists. This knowledge could be leveraged to develop highly targeted training programmes that focus specifically on the identified neuromuscular and kinematic adaptations. For example, training swimmers with parameters similar to those found in pre-mechanical failure may enable them to improve endurance by delaying the onset of mechanical failure as much as possible.

In order to substantiate and further clarify these findings, it is advisable to carry out further studies with a larger sample size. This should include a comparison with sprint specialists and bilateral EMG analysis of the body to provide a more complete and accurate picture of the neuromuscular strategies employed.

## Figures and Tables

**Table 1:** Physical and athletic characteristics of recruited participants. Values are presented as means and standard deviations. Abbreviation: BMI = Body Mass Index; MDS = middle-distance swimmers; LDS = long-distance swimmers; WAPS = World Aquatic Points Scoring (points were based on the swimmers’ best official performance on the 1500 m long course).

Sex	n	Age (Years)	Height (cm)	Body Mass (kg)	BMI (kg/m^2^)	International Competition Experience (Years)	WAPS
Male	15	19.07 ± 2.31	181 73 ± 5.06	73.00 ± 5.55	22.11 ± 1.54	3.87 ± 0.74	795.07 ± 41.39
Female	6	20.83 ± 3.19	170.83 ± 5.49	60.00 ± 4.56	20.53 ± 0.36	4.67 ± 1.75	744.33 ± 66.63
MDS	9	20.11 ± 3.14	182.33 ± 2.25	65.33 ± 6.60	21.72 ± 1.32	4.33 ± 1.50	775.89 ± 63.35
LDS	12	19.16 ± 2.25	173.67 ± 6.60	72.25 ± 5.97	21.58 ± 1.77	3.92 ± 0.79	784 08 ± 47.44
All	21	19.57 ± 2.64	178.62 ± 7.14	69.29 ± 7.94	21.66 ± 1.49	4.10 ± 1.14	780.57 ± 53.48

**Table 2:** Kinematic parameters in three different conditions (non-fatigue, pre-mechanical failure, and mechanical failure). Abbreviations: ES = effect size.

Kinematic Parameters	Non-Fatigue vs. Pre-Mechanical Failure			Non-Fatigue vs. Mechanical Failure			Overall
	Percentage Difference (%)	ES	*p* Value	Percentage Difference (%)	ES	*p* Value	ES
Stroke frequency	7	−0.931	<0.001	−5	0.883	<0.001	0.587
Stroke length	−6	0.948	<0.001	−6	0.827	<0.001	0.403
Stroke index	−6	0.965	<0.001	−15	1.000	<0.001	0.649

**Table 3:** EMG activity for the ten studied muscles in three different conditions (non-fatigue, pre-mechanical failure, and mechanical failure). Abbreviations: E&G = entry and glide; ES = effect size.

		Non-Fatigue vs. Pre-Mechanical Failure			Non-Fatigue vs. Mechanical Failure		
Muscle		Percentage Difference (%)	ES	*p* Value	Percentage Difference (%)	ES	*p* Value
	E&G	16	−1.000	<0.001	8	−0.350	0.252
FRC	PULL	16	−0.961	<0.001	−11	1.000	<0.001
	PUSH	23	−1.000	<0.001	−10	0.448	0.017
	RECOVERY	30	−1.000	<0.001	−11	0.547	<0.001
	E&G	110	−1.000	<0.001	27	−0.450	0.135
BB	PULL	27	−1.000	<0.001	−20	1.000	<0.001
	PUSH	0	0.016	0.941	−17	0.482	0.010
	RECOVERY	18	−0.425	<0.001	0	0.041	0.719
	E&G	13	−1.000	<0.001	−28	1.000	<0.001
TB	PULL	15	−0.866	<0.001	−11	0.888	<0.001
	PUSH	1	−0.286	0.133	−15	0.792	<0.001
	RECOVERY	18	−0.773	<0.001	−34	0.936	<0.001
	E&G	10	−0.933	<0.001	−48	1.000	<0.001
DL	PULL	−11	0.492	0.002	−38	0.689	<0.001
	PUSH	42	−0.855	<0.001	35	−0.872	<0.001
	RECOVERY	13	−0.631	<0.001	−22	0.908	<0.001
	E&G	5	−0.150	0.639	−32	1.000	<0.001
LD	PULL	7	−0.621	<0.001	−8	0.721	<0.001
	PUSH	−17	0.906	<0.001	−13	0.488	0.009
	RECOVERY	51	1.000	<0.001	−18	0.749	<0.001
	E&G	7	−0.883	0.001	−44	1.000	<0.001
ST	PULL	43	−1.000	<0.001	−17	1.000	<0.001
	PUSH	23	−1.000	<0.001	−3	0.016	0.941
	RECOVERY	−2	0.098	0.388	−30	1.000	<0.001
	E&G	−10	0.677	0.022	−14	1.000	<0.001
ES	PULL	13	−0.905	<0.001	11	−0.552	<0.001
	PUSH	0	−0.004	0.988	−25	1.000	<0.001
	RECOVERY	25	−0.671	<0.001	−8	0.144	0.205
	E&G	135	−1.000	<0.001	−13	0.850	0.002
PM	PULL	49	−0.946	<0.001	−11	0.624	<0.001
	PUSH	4	−0.141	0.464	−20	0.553	0.003
	RECOVERY	7	−0.392	<0.001	33	−0.578	<0.001
	E&G	−2	0.467	0.121	−10	0.933	<0.001
RF	PULL	34	−1.000	<0.001	2	−131	0.417
	PUSH	29	−0.741	<0.001	12	−0.974	<0.001
	RECOVERY	29	−0.965	<0.001	4	−0.299	0.008
	E&G	96	−1.000	<0.001	69	−1.000	<0.001
BF	PULL	11	−0.606	<0.001	13	−0.409	0.011
	PUSH	10	−0.553	0.003	4	−0.169	0.378
	RECOVERY	18	0.751	<0.001	4	−0.111	0.329

**Table 4:** Comparison of EMG activity and kinematic parameters across genders and distances in swimming during non-fatigue and pre-mechanical failure phases. Abbreviations: MDS = middle-distance swimmers; LDS = long-distance swimmers; ES = effect size; FCR = *Flexor Carpi Radialis*; BB = *Biceps Brachii*; TB = *Triceps Brachii caput lateralis*; DL = *Deltoideus Lateralis*; LD = *Latissimus Dorsi*; ST = *Superior Trapezius*; ES = *Erector Spinae*; PM = *Pectoralis Major pars clavicularis*; RF = *Rectus Femoris*; BF = *Biceps Femoris*.

	Male vs. Female		MDS vs. LDS	
	Percentage Difference (%)	*p* Value	Percentage Difference (%)	*p* Value
FCR	−1	0.779	3	0.556
BB	−3	0.694	5	0.479
TB	1	0.896	5	0.408
DL	−2	0.869	3	0.811
LD	−11	0.311	5	0.678
ST	0	0.963	8	0.311
ES	−11	0.116	3	0.717
PM	−1	0.839	2	0.786
RF	12	0.376	7	0.520
BF	0	0.941	1	0.851
Kinematics	−25	0.785	7	0.413

**Table 5:** Percentage changes in EMG activity and kinematic measures are assessed at four specific points along the distance travelled, namely, 25%, 50%, 75%, and 100% between the non-fatiguing and pre-mechanical failure phases. Abbreviations: FCR = *Flexor Carpi Radialis*; BB = *Biceps Brachii*; TB = *Triceps Brachii caput lateralis*; DL = *Deltoideus Lateralis*; LD = *Latissimus Dorsi*; ST = *Superior Trapezius*; ES = *Erector Spinae*; PM = *Pectoralis Major pars clavicularis*; RF = *Rectus Femoris*; BF = *Biceps Femoris*; SF = stroke frequency; SL = stroke length.

	Distance Travelled between Non-Fatigue and Pre-Mechanical Failure			
	25%	50%	75%	100%
*Change in EMG activity (%)*				
FCR	3.05 ± 1.54	7.68 ± 0.86	10.99 ± 2.97	15.35 ± 1.72
BB	1.77 ± 0.71	3.44 ± 0.52	4.79 ± 1.03	6.88 ± 1.04
TB	1.50 ± 0.64	2.71 ± 0.38	3.59 ± 0.77	5.42 ± 0.76
DL	2.36 ± 4.31	2.98 ± 0.68	4.07 ± 1.39	5.95 ± 1.37
LD	1.92 ± 1.01	4.13 ± 1.02	5.09 ± 1.71	8.27 ± 2.05
ST	1.77 ± 0.72	4.07 ± 0.65	5.96 ± 1.03	8.14 ± 1.29
ES	1.26 ± 1.03	1.37 ± 0.22	2.24 ± 0.58	2.75 ± 0.43
PM	1.78 ± 0.79	3.86 ± 0.51	5.16 ± 1.15	7.72 ± 1.02
RF	1.79 ± 0.88	4.16 ± 1.08	5.67 ± 1.41	8.32 ± 2.16
BF	1.52 ± 0.91	4.23 ± 0.40	6.11 ± 1.07	8.46 ± 0.81
*Change in kinematics (%)*				
SF	1.51 ± 0.64	3.15 ± 1.28	4.52 ± 1.91	6.12 ± 2.55
SL	−1.51 ± 0.64	−3.15 ± 1.28	−4.52 ± 1.91	−6.12 ± 2.55

## Data Availability

All data generated are within the main text. Further data can be obtained upon request to the corresponding author.

## References

[1] Cifrek M., Medved V., Tonković S., Ostojić S. (2009). Surface EMG Based Muscle Fatigue Evaluation in Biomechanics. Clin. Biomech..

[2] Behm D.G. (2004). Force Maintenance with Submaximal Fatiguing Contractions. Can. J. Appl. Physiol..

[3] Bigland-Ritchie B., Woods J.J. (1984). Changes in Muscle Contractile Properties and Neural Control during Human Muscular Fatigue. Muscle Nerve.

[4] Gandevia S.C. (2001). Spinal and Supraspinal Factors in Human Muscle Fatigue. Physiol. Rev..

[5] Hainaut K., Duchateau J. (1989). Muscle fatigue, effects of training and disuse. Muscle Nerve.

[6] Kouzaki M., Shinohara M. (2006). The Frequency of Alternate Muscle Activity Is Associated with the Attenuation in Muscle Fatigue. J. Appl. Physiol..

[7] Edwards R.G., Lippold O.C. (1956). The Relation between Force and Integrated Electrical Activity in Fatigued Muscle. J. Physiol..

[8] Bigland-Ritchie B., Johansson R., Lippold O.C., Woods J.J. (1983). Contractile Speed and EMG Changes during Fatigue of Sustained Maximal Voluntary Contractions. J. Neurophysiol..

[9] Turpin N.A., Guével A., Durand S., Hug F. (2011). Fatigue-Related Adaptations in Muscle Coordination during a Cyclic Exercise in Humans. J. Exp. Biol..

[10] Billaut F., Basset F.A., Falgairette G. (2005). Muscle Coordination Changes during Intermittent Cycling Sprints. Neurosci. Lett..

[11] Dorel S., Drouet J.M., Couturier A., Champoux Y., Hug F. (2009). Changes of Pedaling Technique and Muscle Coordination during an Exhaustive Exercise. Med. Sci. Sports Exerc..

[12] Bonnard M., Sirin A.V., Oddsson L., Thorstensson A. (1994). Different Strategies to Compensate for the Effects of Fatigue Revealed by Neuromuscular Adaptation Processes in Humans. Neurosci. Lett..

[13] Rodacki A.L.F., Fowler N.E., Bennett S.J. (2002). Vertical Jump Coordination: Fatigue Effects. Med. Sci. Sports. Exerc..

[14] Vaz J.R., Olstad B.H., Cabri J., Kjendlie P.-L., Pezarat-Correia P., Hug F. (2016). Muscle Coordination during Breaststroke Swimming: Comparison between Elite Swimmers and Beginners. J. Sports Sci..

[15] Matsuura Y., Matsunaga N., Akuzawa H., Kojima T., Oshikawa T., Iizuka S., Okuno K., Kaneoka K. (2022). Difference in Muscle Synergies of the Butterfly Technique with and without Swimmer’s Shoulder. Sci. Rep..

[16] Matsuura Y., Matsunaga N., Iizuka S., Akuzawa H., Kaneoka K. (2020). Muscle Synergy of the Underwater Undulatory Swimming in Elite Male Swimmers. Front. Sports Act. Living.

[17] Matsuura Y., Matsunaga N., Akuzawa H., Oshikawa T., Kaneoka K. (2023). Comparison of Muscle Coordination during Front Crawl and Backstroke with and without Swimmer’s Shoulder Pain. Sports Health.

[18] Martens J., Figueiredo P., Daly D. (2015). Electromyography in the Four Competitive Swimming Strokes: A Systematic Review. J. Electromyogr. Kinesiol..

[19] Deschodt J.V., Arsac L.M., Rouard A.H. (1999). Relative Contribution of Arms and Legs in Humans to Propulsion in 25-m Sprint Front-Crawl Swimming. Eur. J. Appl. Physiol. Occup. Physiol..

[20] Aujouannet Y.A., Bonifazi M., Hintzy F., Vuillerme N., Rouard A.H. (2006). Effects of a High-Intensity Swim Test on Kinematic Parameters in High-Level Athletes. Appl. Physiol. Nutr. Metab..

[21] Seifert L., Chollet D., Rouard A. (2007). Swimming Constraints and Arm Coordination. Hum. Mov. Sci..

[22] Craig A.B., Skehan P.L., Pawelczyk J.A., Boomer W.L. (1985). Velocity, stroke rate, and distance per stroke during elite swimming competition. Med. Sci. Sports Exerc..

[23] Pai Y., Hay J.G., Wilson B.D. (1984). Stroking Techniques of Elite Swimmers. J. Sports Sci..

[24] Alberty M., Sidney M., Huot-Marchand F., Hespel J.M., Pelayo P. (2005). Intracyclic Velocity Variations and Arm Coordination during Exhaustive Exercise in Front Crawl Stroke. Int. J. Sports Med..

[25] Alberty M., Potdevin F., Dekerle J., Pelayo P., Gorce P., Sidney M. (2008). Changes in Swimming Technique during Time to Exhaustion at Freely Chosen and Controlled Stroke Rates. J. Sports Sci..

[26] Figueiredo P., Vilas-Boas J.P., Seifert L., Chollet D., Fernandes R.J. (2014). Inter-Limb Coordinative Structure in a 200 m Front Crawl Event. Open Sports Sci. J..

[27] Seifert L., Chollet D., Bardy B. (2004). Effect of Swimming Velocity on Arm Coordination in the Front Crawl: A Dynamic Analysis. J. Sports Sci..

[28] Kwok W.Y., So B.C.L., Ng S.M.S. (2022). Underwater Surface Electromyography for the Evaluation of Muscle Activity during Front Crawl Swimming: A Systematic Review. J. Sports Sci. Med..

[29] Ikuta Y., Matsuda Y., Yamada Y., Kida N., Oda S., Moritani T. (2012). Relationship between Decreased Swimming Velocity and Muscle Activity during 200-m Front Crawl. Eur. J. Appl. Physiol..

[30] Stirn I., Jarm T., Kapus V., Strojnik V. (2011). Evaluation of Muscle Fatigue during 100-m Front Crawl. Eur. J. Appl. Physiol..

[31] Figueiredo P., Rouard A., Vilas-Boas J.P., Fernandes R.J. (2013). Upper- and Lower-Limb Muscular Fatigue during the 200-m Front Crawl. Appl. Physiol. Nutr. Metab..

[32] Figueiredo P., Sanders R., Gorski T., Vilas-Boas J., Fernandes R. (2012). Kinematic and Electromyographic Changes during 200 m Front Crawl at Race Pace. Int. J. Sports Med..

[33] Conceição A., Silva A.J., Barbosa T., Karsai I., Louro H. (2014). Neuromuscular Fatigue during 200 M Breaststroke. J. Sports Sci. Med..

[34] Lomax M., Tasker L., Bostanci O. (2014). Inspiratory Muscle Fatigue Affects Latissimus Dorsi but Not Pectoralis Major Activity during Arms Only Front Crawl Sprinting. J. Strength Cond. Res..

[35] Puce L., Trompetto C., Currà A., Marinelli L., Mori L., Panascì M., Cotellessa F., Biz C., Bragazzi N.L., Ruggieri P. (2022). The Effect of Verbal Encouragement on Performance and Muscle Fatigue in Swimming. Medicina.

[36] Costill D.L., Kovaleski J., Porter D., Kirwan J., Fielding R., King D. (1985). Energy Expenditure during Front Crawl Swimming: Predicting Success in Middle-Distance Events. Int. J. Sports Med..

[37] Puce L., Chamari K., Marinelli L., Mori L., Bove M., Faelli E., Fassone M., Cotellessa F., Bragazzi N.L., Trompetto C. (2022). Muscle Fatigue and Swimming Efficiency in Behind and Lateral Drafting. Front. Physiol..

[38] Rainoldi A., Cescon C., Bottin A., Casale R., Caruso I. (2004). Surface EMG Alterations Induced by Underwater Recording. J. Electromyogr. Kinesiol..

[39] Ryan M.M., Gregor R.J. (1992). EMG Profiles of Lower Extremity Muscles during Cycling at Constant Workload and Cadence. J. Electromyogr. Kinesiol..

[40] Puce L., Pallecchi I., Marinelli L., Mori L., Bove M., Diotti D., Ruggeri P., Faelli E., Cotellessa F., Trompetto C. (2021). Surface Electromyography Spectral Parameters for the Study of Muscle Fatigue in Swimming. Front. Sports Act. Living.

[41] Tomczak M., Tomczak E. (2014). The need to report effect size estimates revisited. An overview of some recommended measures of effect size. Trends Sport Sci..

[42] Bakker A., Cai J., English L., Kaiser G., Mesa V., Van Dooren W. (2019). Beyond Small, Medium, or Large: Points of Consideration When Interpreting Effect Sizes. Educ. Stud. Math..

[43] López-Martín E., Ardura D. (2023). The effect size in scientific publication. Educación XX1.

[44] Cureton E.E. (1956). Rank-biserial correlation. Psychometrika.

[45] Hirai H., Miyazaki F., Naritomi H., Koba K., Oku T., Uno K., Uemura M., Nishi T., Kageyama M., Krebs H.I. (2015). On the Origin of Muscle Synergies: Invariant Balance in the Co-Activation of Agonist and Antagonist Muscle Pairs. Front. Bioeng. Biotechnol..

[46] Rouard A., Billat R. (1990). Influences of Sex and Level of Performance on Freestyle Stroke: An Electromyography and Kinematic Study. Int. J. Sports Med..

[47] Pink M., Perry J., Browne A., Scovazzo M.L., Kerrigan J. (1991). The Normal Shoulder during Freestyle Swimming: An Electromyographic and Cinematographic Analysis of Twelve Muscles. Am. J. Sports Med..

[48] Rouard A.H., Billat R.P., Deschodt V., Clarys J.P. (1997). Muscular Activations during Repetitions of Sculling Movements up to Exhaustion in Swimming. Arch. Physiol. Biochem..

[49] Caty V., Aujouannet Y., Hintzy F., Bonifazi M., Clarys J.P., Rouard A.H. (2007). Wrist stabilisation and forearm muscle coactivation during freestyle swimming. J. Electromyogr. Kinesiol..

[50] Petrofsky J.S. (1979). Frequency and Amplitude Analysis of the EMG during Exercise on the Bicycle Ergometer. Eur. J. Appl. Physiol. Occup. Physiol..

[51] González-Izal M., Malanda A., Gorostiaga E., Izquierdo M. (2012). Electromyographic Models to Assess Muscle Fatigue. J. Electromyogr. Kinesiol..

[52] Henneman E., Somjen G., Carpenter D.O. (1965). Excitability and Inhibitibility of Motoneurons of Different Sizes. J. Neurophysiol..

[53] Enoka R.M., Duchateau J. (2016). Translating Fatigue to Human Performance. Med. Sci. Sports Exerc..

[54] Laudner K.G., Williams J.G. (2013). The Relationship between Latissimus Dorsi Stiffness and Altered Scapular Kinematics among Asymptomatic Collegiate Swimmers. Phys. Ther. Sport.

[55] Payton C., Hogarth L., Burkett B., Van De Vliet P., Lewis S., Oh Y.-T. (2020). Active Drag as a Criterion for Evidence-Based Classification in Para Swimming. Med. Sci. Sports Exerc..

[56] Toussaint H.M., Beek P.J. (1992). Biomechanics of Competitive Front Crawl Swimming. Sports Med..

[57] Pollock S., Gaoua N., Johnston M.J., Cooke K., Girard O., Mileva K.N. (2019). Training Regimes and Recovery Monitoring Practices of Elite British Swimmers. J. Sports Sci. Med..

[58] Hermosilla F., González-Rave J.M., Del Castillo J.A., Pyne D.B. (2021). Periodization and Programming for Individual 400 m Medley Swimmers. Int. J. Environ. Res. Public Health.

[59] Lepers R., Maffiuletti N.A. (2011). Age and gender interactions in ultraendurance performance: Insight from the triathlon. Med. Sci. Sports Exerc..

[60] Lepers R. (2008). Analysis of Hawaii ironman performances in elite triathletes from 1981 to 2007. Med. Sci. Sports Exerc..

[61] Tanaka H., Seals D.R. (1997). Age and gender interactions in physiological functional capacity: Insight from swimming performance. J. Appl. Physiol..

[62] Vogt P., Rüst C.A., Rosemann T., Lepers R., Knechtle B. (2013). Analysis of 10 km swimming performance of elite male and female open-water swimmers. SpringerPlus.

